# Clinical Determinants and Barriers to Cardiac Rehabilitation Enrollment of Patients with Heart Failure with Reduced Ejection Fraction: A Single-Center Study in Portugal

**DOI:** 10.3390/jcdd9100344

**Published:** 2022-10-09

**Authors:** André Alexandre, Cristine Schmidt, Andreia Campinas, Catarina Gomes, Sandra Magalhães, José Preza-Fernandes, Severo Torres, Mário Santos

**Affiliations:** 1 Department of Cardiology, Centro Hospitalar Universitário do Porto (CHUPorto), 4099-001 Porto, Portugal; 2 ICBAS—School of Medicine and Biomedical Sciences, University of Porto, 4050-313 Porto, Portugal; 3 Department of Physiology and Cardiothoracic Surgery, Faculty of Medicine, University of Porto, 4200-319 Porto, Portugal; 4 CIAFEL—Physical Activity, Health and Leisure Research Centre, Faculty of Sports, University of Porto, 4200-450 Porto, Portugal; 5 ITR—Laboratory for Integrative and Translational Research in Population Health, 4050-600 Porto, Portugal; 6 Unit of Cardiovascular Prevention and Rehabilitation, Centro Hospitalar Universitário do Porto (CHUPorto), 4099-001 Porto, Portugal; 7 Department of Physical and Rehabilitation Medicine, Centro Hospitalar Universitário do Porto (CHUPorto), 4099-001 Porto, Portugal; 8 UMIB—Unit for Multidisciplinary Research in Biomedicine, ICBAS—School of Medicine and Biomedical Sciences, University of Porto, 4050-313 Porto, Portugal

**Keywords:** cardiac rehabilitation, barriers, clinical determinants, heart failure, reduced ejection fraction

## Abstract

Despite cardiac rehabilitation (CR) being a recommended treatment for patients with heart failure with reduced ejection fraction (HFrEF), it is still underused. This study investigated the clinical determinants and barriers to enrollment in a CR program for HFrEF patients. We conducted a cohort study using the Cardiac Rehabilitation Barriers Scale (CRBS) to assess the reason for non-enrollment. Of 214 HFrEF patients, 65% had not been enrolled in CR. Patients not enrolled in CR programs were older (63 vs. 58 years; *p* < 0.01) and were more likely to have chronic obstructive pulmonary disease (COPD) (20% vs. 5%; *p* < 0.01). Patients enrolled in CR were more likely to be treated with sacubitril/valsartan (34% vs. 19%; *p* = 0.01), mineralocorticoid receptor antagonists (84% vs. 72%; *p* = 0.04), an implantable cardioverter defibrillator (ICD) (41% vs. 20%; *p* < 0.01), and cardiac resynchronization therapy (21% vs. 10%; *p* = 0.03). Multivariate analysis revealed that age (adjusted OR 1.04; 95% CI 1.01–1.07), higher education level (adjusted OR 3.31; 95% CI 1.63–6.70), stroke (adjusted OR 3.29; 95% CI 1.06–10.27), COPD (adjusted OR 4.82; 95% CI 1.53–15.16), and no ICD status (adjusted OR 2.68; 95% CI 1.36–5.26) were independently associated with CR non-enrollment. The main reasons for not being enrolled in CR were no medical referral (31%), concomitant medical problems (28%), patient refusal (11%), and geographical distance to the hospital (9%). Despite the relatively high proportion (35%) of HFrEF patients who underwent CR, the enrollment rate can be further improved. Innovative multi-level strategies addressing physicians’ awareness, patients’ comorbidities, and geographical issues should be pursued.

## 1. Introduction

Heart failure with reduced ejection fraction (HFrEF) is a leading cause of morbidity and mortality worldwide [1]. Despite the improvement of its prognosis in the last decades, HFrEF patients still face high mortality rates and reduced quality of life (QOL) [1]. Cardiac rehabilitation (CR) is a comprehensive program that includes exercise training, secondary prevention, and lifestyle changes, which can lessen the burden of HFrEF [2,3,4,5]. There is consistent evidence that CR improves exercise tolerance and health-related QOL in patients with heart failure [1]. Meta-analyses of randomized clinical trials studying the impact of CR in HFrEF patients show a significant reduction in hospitalizations [1,2,3]. Therefore, international guidelines strongly recommend CR for patients with HFrEF [1,3,6,7,8,9]. Nevertheless, despite the robust evidence supporting its safety and benefits, CR programs are underutilized by this high-risk population [2,10,11,12,13,14,15,16,17,18,19]. It is theorized that the reasons why this happens are complex, heterogeneous, and encompass healthcare system-, referring physician-, program-, and patient-level barriers [2,12]. There are very few studies that consider these multi-level barriers concurrently [2,3]. The main purpose of this study is to investigate the clinical determinants and barriers to CR enrollment of HFrEF patients in a tertiary hospital in Portugal. Our hypothesis is that barriers to participation in a CR program would be equally related to healthcare system-, referring physician-, and patient-levels.

## 2. Methods

### 2.1. Design and Procedure

We conducted a retrospective cohort study of 236 consecutive heart failure patients at a HFrEF cardiology outpatient clinic from January 2019 to April 2021. The study population was defined as patients with a confirmed diagnosis of HFrEF according to criteria of the European Society of Cardiology (ESC) [1], who had a clinic visit between January 2019 to April 2021, and who were eligible to participate in CR based on CR guidelines [4,6,7,8,9]. Patients without confirmed HFrEF were excluded from the analysis (*n* = 8). Patients with HFrEF and documentation of acute myocardial infarction in the previous 12 months were also excluded (*n* = 14), as they might have been enrolled in a CR program due to acute coronary syndrome. The remaining 214 HFrEF patients included in the study were divided into two groups according to their participation in a CR program: patients enrolled in CR versus patients not enrolled in CR. Of note, patients with previous CR over 12 months ago for any other indication were not excluded from the analysis, and they were also divided into two groups (patients enrolled in CR versus patients not enrolled in CR) according to participation in a CR program specifically for HFrEF. For those who did not participate in a CR program, we classified the reason according to the Cardiac Rehabilitation Barriers Scale (CRBS; a structured and validated questionnaire for this purpose), which consists of five subscales: comorbidities/functional status, lack of perceived need, personal/family issues, travel/work conflicts, and access [2,5]. If the data from electronic health records were missing or difficult to interpret, CRBS was applied by a phone call to the patients. The CRBS was developed and psychometrically validated by Shanmugasegaram and colleagues in English [5]. It was later translated, culturally adapted, and psychometrically validated in Brazilian Portuguese, the version we used [20]. In item 21 of the CRBS (“Other reason(s) for not attending a cardiac rehabilitation program”), we did an open question to understand it better, and some of our patients referred to the following reasons: “COVID-19 pandemic”, “concomitant respiratory rehabilitation”, and “patient refusal—no reason” [21]. In our study, low education level was defined as completion of education to primary school level or below (≤4th grade) [10].

### 2.2. Statistical Analyses

Statistical analysis was performed using the IBM software Statistical Package for Social Sciences (SPSS) version 26.0. Continuous variables were presented as means and standard deviations (SD) for normally distributed variables, and medians and interquartile ranges for non-normally distributed variables. Categorical variables were reported by numbers and proportions (%). Comparisons between CR program participants versus CR program non-participants were carried out for each individual variable using Student’s T-test or the Mann–Whitney U test for continuous variables, and the chi-squared test or Fisher’s exact test for categorical variables as appropriate. The correlation between variables and CR enrollment was assessed by univariate and multivariate logistic regression. Variables with *p*-value < 0.10 in univariate analysis entered the multivariate stage. Regression analysis results are reported as odds ratio (OR) and 95% confidence intervals (CI). A two-sided *p*-value < 0.05 was considered as indicating statistical significance.

### 2.3. Ethical Approval

The study was approved by the Ethics Committee of Centro Hospitalar Universitário do Porto (CHUPorto) and Instituto de Ciências Biomédicas Abel Salazar (ICBAS) [2019/123(103-DEFI/107-CE)], and it was conducted in accordance with the Declaration of Helsinki.

## 3. Results

### 3.1. Baseline Demographic and Clinical Characteristics

Of the 236 heart failure patients followed at a dedicated HFrEF cardiology clinic from January 2019 to April 2021, a total of 214 patients with HFrEF were included in our study. The majority of patients were male (73%), and the average age was 61 ± 11 years. Regarding CR program participation, 35% (*n* = 76) of patients with HFrEF were enrolled in CR, while 65% (*n* = 138) were not.

Table 1 shows the baseline demographic and clinical characteristics of the studied population according to participation in a CR program. Patients not enrolled in CR were older (63 vs. 58 years; *p* = 0.003) than patients enrolled in CR programs. There were no differences by sex (70% vs. 79%; *p* = 0.170). Patients enrolled in CR were more likely to have a lower education level when compared to patients not participating in CR (38% vs. 18%; *p* = 0.001).

Patients not enrolled in CR programs were more likely to have chronic obstructive pulmonary disease [COPD] (20% vs. 5%; *p* = 0.004). There was also a trend for patients not enrolled in CR to have a history of stroke (15% vs. 7%; *p* = 0.085) or hypertension (56% vs. 42%; *p* = 0.055). There were no differences between groups regarding diabetes mellitus, dyslipidemia, smoking, body mass index, previous acute myocardial infarction, known coronary artery disease, peripheral artery disease, atrial fibrillation, chronic kidney disease, or cancer.

### 3.2. Heart Failure-Related Features

We did not find any significant differences between groups regarding heart failure-related clinical features, such as etiology of heart failure, New York Heart Association (NYHA) functional class, N-terminal pro-B-type natriuretic peptide (NT-pro-BNP) levels, left ventricular ejection fraction (LVEF), pulmonary artery systolic pressure (PASP), or mitral regurgitation severity on echocardiography. They are displayed in Table 2.

Patients enrolled in CR programs were more likely treated with sacubitril/valsartan (34% vs. 19%; *p* = 0.012) and with mineralocorticoid receptor antagonists (84% vs. 72%; *p* = 0.040). There were no differences in other pharmacologic therapies between groups (Table 3).

When considering cardiac implantable electronic devices (CIED), patients enrolled in CR programs were more likely to have an implantable cardioverter defibrillator (ICD) (41% vs. 20%; *p* = 0.001) and a cardiac resynchronization therapy (CRT) device (21% vs. 10%; *p* = 0.028), as shown in Table 3.

### 3.3. Univariate Analysis

The correlation between variables and CR enrollment was assessed by univariate logistic regression. The clinical determinants and respective crude odds ratio (OR) of non-enrollment in CR program are shown in Table 4.

### 3.4. Multivariate Analysis

Multivariate analysis revealed that age, education level, stroke, COPD, and no ICD status were independently associated with CR non-enrollment (Table 5).

### 3.5. Barriers to Cardiac Rehabilitation

The barriers to CR enrollment of patients with HFrEF according to the CRBS questionnaire are shown in Table 6. The main reasons for not being enrolled in CR programs were no medical referral (“my doctor did not feel it was necessary”—31%), concomitant medical problems (“other health problems prevent me from going”—28%), patient refusal (“no reason”—11%), and geographical distance to the hospital (“because of distance”—9%).

**Figure 1:**
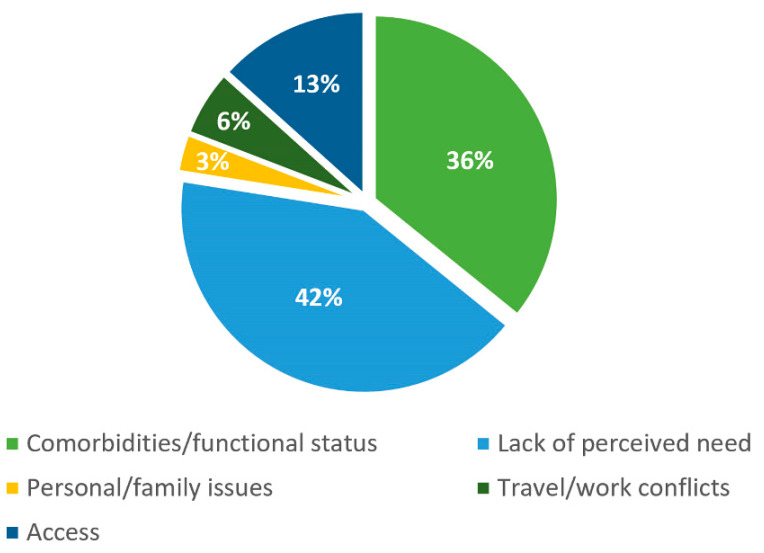
Distribution of barriers to cardiac rehabilitation (CR) enrollment of patients with heart failure with reduced ejection fraction (HFrEF) according to the five subscales of CRBS*: comorbidities/functional status (items 8, 9, 13, 14, 15, 17, 21), lack of perceived need (items 3, 5, 6, 11, 16), personal/family issues (items 4, 7, 18), travel/work conflicts (items 10, 12), and access (items 1, 2, 19, 20). *Item 22 (Other reason) is not classified. CR: cardiac rehabilitation; CRBS: Cardiac Rehabilitation Barriers Scale; HFrEF: heart failure with reduced ejection fraction.

Figure 1 shows the distribution of barriers to CR enrollment of patients with HFrEF according to the five subscales of CRBS: comorbidities/functional status, lack of perceived need, personal/family issues, travel/work conflicts, and access [2]. Lack of perceived need was the predominant subscale (42%). Comorbidities/functional status was the second most important subscale of barriers (36%).

**Figure 2:**
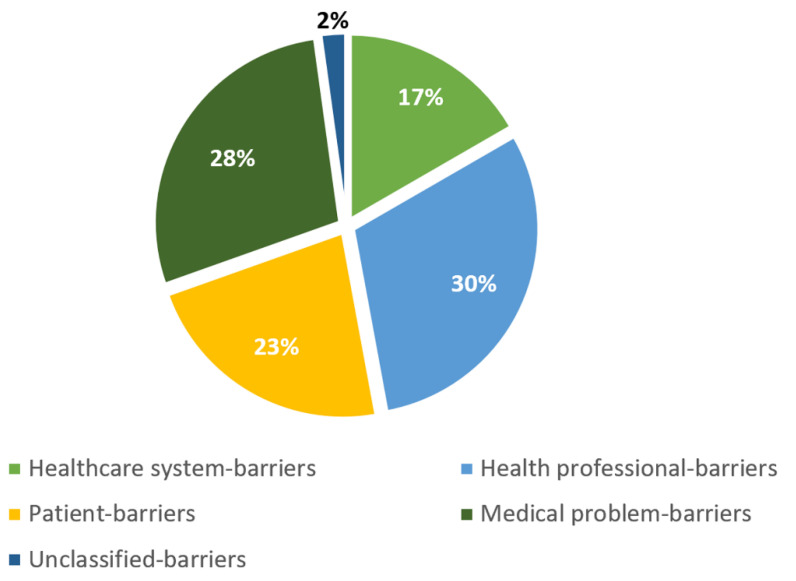
Levels of barriers to cardiac rehabilitation (CR) enrollment. Distribution of barriers to cardiac rehabilitation (CR) enrollment of patients with heart failure with reduced ejection fraction (HFrEF) by different levels: healthcare system-level (items 1, 2, 3, 5, 19, 20), health professional-level (item 16), patient-level (items 4, 6, 7, 8, 9, 10, 11, 12, 13, 15, 17, 18, 21, 22.3), medical problem-level (item 14), and unclassified-level (items 22.1, 22.2). CR: cardiac rehabilitation; HFrEF: heart failure with reduced ejection fraction.

Figure 2 shows the distribution of barriers according to different levels: healthcare system-, health professional-, patient-, medical problem-, and unclassified-level.

## 4. Discussion

This is the first study to investigate the clinical determinants and barriers to enrollment in a CR program for HFrEF patients in a tertiary hospital in Portugal. Regarding the situation of CR in Portugal and according to Fontes et al. [22], 25 centers (16 public, 9 private) provide structured CR programs nationwide. All centers reported multidisciplinary teams, all of which included a cardiologist [22]. Regarding the number of participants, in 2019, a total of 2182 patients underwent phase II programs [22]. Of these, 67.2% were referred due to ischemic heart disease, and 14.5% due to heart failure [22].

Despite CR being a recommended treatment for patients with HFrEF, the majority of studies about CR refer to patients with ischemic heart disease. Indeed, there are very few studies concerning CR enrollment rates in HFrEF. According to Thomas et al. [13] from Mayo Clinic, Rochester (Minnesota–USA), only about 20–30% of patients who are eligible for CR are actually referred to and enroll in CR. Rengo et al. [14] found that only 17% of HFrEF inpatients attended CR in a study conducted in Burlington (Vermont–USA). Unfortunately, the authors were unable to determine the denominator of HFrEF patients seen in outpatient clinics and thus could not calculate overall attendance rates [14]. In a cohort of more than 397,000 Medicare beneficiaries in the USA hospitalized with heart failure who were eligible for CR in 2017, only 2.6% completed ≥1 CR session sometime during the subsequent 12 months; in these CR patients the average number of sessions completed was 22, and 20% completed all 36 allowable sessions [15]. A European survey showed that <20% of patients with heart failure are involved in cardiac rehabilitation [16]. According to Dalal et al. [17], patients with heart failure as a primary diagnosis are excluded from most cardiac rehabilitation programs in England, Wales, and Northern Ireland; the main barriers are a lack of resources and direct exclusion from local commissioning agreements. In the study of Kamiya et al. [18], only 26% of patients hospitalized for acute heart failure at 15 hospitals in Japan participated in outpatient CR. Also, very few patients with heart failure (7.3%, 3741/51,323 patients) received outpatient CR [19]. All these studies demonstrate that CR is still underused in heart failure patients.

Our research contributes valuable insights regarding clinical determinants and barriers to CR enrollment of HFrEF patients in a tertiary hospital in Portugal. We found that 35% of eligible HFrEF patients were enrolled in a CR program, which is a relatively higher proportion compared to the aforementioned studies. Yet, the fact that 65% of patients were not enrolled in CR highlights that the enrollment rate can be further improved and that the main barriers to enrollment must be identified and properly targeted.

In general, our data demonstrated that patients not enrolled in CR programs were older and were more likely to have comorbidities, such as stroke or COPD. Multivariate analysis revealed that age, education level, stroke, COPD, and no ICD status were independently associated with CR non-enrollment. Our research also revealed that the main reasons for not being enrolled in CR programs were no medical referral, concomitant medical problems, patient refusal, and geographical distance to the hospital.

Similar to other studies [10,23,24,25,26,27,28,29], we found that patients not enrolled in CR programs were older and more likely to have comorbidities (such as stroke or COPD). In our study, age remained significantly associated with CR non-enrollment after adjusting for confounding variables. Indeed, according to Ruano-Ravina et al. [28] and Neubeck et al. [29], older age is a barrier to participation in CR, which declines significantly after age 70 [30]. One possible reason is that older patients may perceive themselves as being less in control of their illness and therefore less likely to participate in CR programs [31,32].

Unlike most previous studies that report that women are significantly less likely to access CR than men [23,27,28,29,33,34,35,36,37,38], we found no differences in CR program enrollment between men and women.

Surprisingly in our cohort population, patients enrolled in CR programs were more likely to have lower education levels (defined as completion of education to primary school level or below), which differs from previous studies [10,38]. This can be due to chance because of the categorization employed, since the group of patients who schooled for more than 4 years is quite heterogeneous. However, it can also reflect different degrees of employability, which hamper the participation of patients with higher education levels in CR programs that occur during working hours. More granular data on the conflict between work and CR programs should be gathered in future studies.

In our research, the data presented suggest patients being referred to CR are better treated regarding optimal medical treatment and devices. Indeed, patients enrolled in CR programs were more likely treated with sacubitril/valsartan and with mineralocorticoid receptor antagonists, but not with beta-blockers, ACE-I/ARB, SGLT2-I, or loop diuretics. On the other hand, multivariate analysis did not show sacubitril/valsartan and mineralocorticoid receptor antagonists were independently associated with CR enrollment. Hence, this finding is possibly related to CR implementation itself (and not a clinical determinant of CR participation) since CR provides an opportunity to address medication optimization and adherence [39,40]. Also, it can be due to sample size as a confounding factor. 

Regarding cardiac implantable electronic devices (CIED), we found that patients enrolled in CR programs were more likely to have ICD and CRT. It is known that despite the fear of shock occurrence during exercise training, supervised exercise testing and training are feasible and safe in a medical environment with a well-trained staff [41,42]. The presence of CIED in a higher proportion of patients enrolled in CR programs could be explained as related to the implementation of CR itself (since it provides an opportunity to optimize heart failure treatment) [39,40]. However, in multivariate analysis, the presence of ICD remained independently associated with CR enrollment, highlighting that these patients may have higher referral rates due to systematic CR referral after device implantation for exercise training instruction [42]. More data on this association should be gathered in future studies.

Regarding barriers to CR enrollment, previous research has consistently identified inadequate referral as a major barrier [24,34]. In our study, lack of referral (“my doctor did not feel it was necessary”) was identified as being responsible for 31% of patients not participating in CR. It is known that referral processes for patients with heart failure are generally underdeveloped compared to other cardiac conditions and are less likely to use automated methods or proactive screening [43]. Accordingly, we think that the main reasons for the lack of recommendation for CR referral in our specialized HFrEF center were time constraints during healthcare visits and a perceived lack of resources for recruiting a larger proportion of patients. Less likely reasons were perceived patient’s disability to participate in CR not written on the medical records, distinct awareness of the CR indication by different physicians on the team, and perception that other healthcare providers should provide this referral. Our research definitely confirms that physician referral remains inadequate. One solution might be systematic CR referral as a possible way to achieve higher referral rates [12,14,24,34,38].

Proximity to a CR center has been found to play an important role in enrollment in CR programs [23]. Brual et al. [44] showed that a driving time of 60 min or more to the nearest CR center was associated with decreased CR referral and enrollment. In our study, geographical distance to the hospital was a major barrier and responsible for 9% of patients not participating in CR. The variable importance of this factor across studies is probably dependent on the geographical dispersion of the population served by each hospital. In an attempt to overcome geographical barriers, the delivery of hybrid and home-based CR, as well as the delivery of CR in community health service centers exploiting existing physical infrastructures (community exercise centers), may increase patients’ enrollment in the future [2,4,45,46,47,48,49]. Besides that, patients who were not enrolled in CR reported concomitant medical problems (28%), namely musculoskeletal problems (15%), as a common barrier to CR. Thus, personalized programs more adapted to these medical limitations could address this major barrier and promote CR adherence [12,50,51,52].

The inter-relationship among barriers at each level is evident in our research. It demonstrates that we can no longer focus solely on patient-level barriers, and we must examine the broader issues affecting CR at healthcare system- and health professional-levels as well. The lack of resources to deliver CR is a major problem. The high cost of CR programs hinders CR implementation, as does the lack of government initiatives to create more CR programs, and the lack of knowledge about CR in the non-medical community [2]. On the other hand, without expanding CR programs to other geographical areas, patients will continue to have barriers related to distance, cost, and transportation. Thus, change must start at the foundation of healthcare systems with the implementation of programs that promote and advocate for more CR services, as this would empower physicians for more practical referrals and limit some patient-level barriers. Studies evaluating the effect of such strategies are scarce, and this represents an important direction for future research [2]. Improving access to CR is crucial and should be given high priority for better care of HFrEF patients.

### Strengths and Limitations

This study has several limitations that need to be considered. First, the study population was derived from a single tertiary academic hospital of a universal coverage healthcare system, which limits the generalizability to different hospitals and healthcare systems. Second, all patients were recruited from a dedicated HFrEF cardiology consult done by HF-dedicated cardiologists, which are more likely to be aware of the benefits of CR programs. Thus, our results might not be generalizable to other HFrEF populations treated by nonspecialized physicians, or to inpatients with acute heart failure. Third, we acknowledge that social determinants of health, such as poverty, were not explicitly addressed in our study, although they are possible confounding factors. Fourth, some barriers to CR enrollment were based on patients’ self-reported information, which is not on an objective measure and can be a limitation in this study. Still, insights into patients’ perspectives are important for a deeper understanding of real aspects that influence participation in CR programs.

## 5. Conclusions

Despite the relatively high proportion (35%) of HFrEF patients who underwent a CR program compared to previous studies, the enrollment rate can be further improved. The main barriers are interdependent and related to health professionals (no referral), the healthcare system (geographical distance to the hospital), and patients (concomitant noncardiac problems). Innovative multi-level strategies addressing these factors should be pursued to increase the delivery of CR programs in HFrEF.

## Figures and Tables

**Table 1:** Baseline demographic and clinical characteristics of the studied population according to participation in a cardiac rehabilitation program.

Demographic Characteristics	Overall (*n* = 214)	Enrolled in CR (*n* = 76)	Not Enrolled in CR (*n* = 138)	*p*-Value
Age (years), mean (SD)	61.3 (11.4)	58.2 (10.2)	63.0 (11.7)	0.003
Men, *n* (%)	157 (73.4)	60 (78.9)	97 (70.3)	0.170
Education level				
≤4th grade, *n* (%) *	54 (25.4)	29 (38.2)	25 (18.2)	0.001
>4th grade, *n* (%) *	159 (74.6)	47 (61.8)	112 (81.8)	0.001
Medical History				
Hypertension, *n* (%)	109 (50.9)	32 (42.1)	77 (55.8)	0.055
Diabetes mellitus, *n* (%) *	75 (35.2)	25 (32.9)	50 (36.5)	0.598
Dyslipidaemia, *n* (%)	135 (63.1)	52 (68.4)	83 (60.1)	0.230
Smoking, *n* (%) *	138 (64.8)	51 (68.0)	87 (63.0)	0.469
BMI (kg/m^2^), mean (SD)	27.7 (6.3)	27.2 (6.2)	28.1 (6.4)	0.538
AMI, *n* (%)	43 (20.1)	14 (18.4)	29 (21.0)	0.650
CAD, *n* (%) ^#^	87 (41.0)	33 (43.4)	54 (39.7)	0.598
PAD, *n* (%)	23 (10.7)	9 (11.8)	14 (10.1)	0.701
Stroke, *n* (%)	25 (11.7)	5 (6.6)	20 (14.5)	0.085
Atrial fibrillation, *n* (%)	59 (27.6)	19 (25.0)	40 (29.0)	0.532
CKD, *n* (%)	9 (4.2)	1 (1.3)	8 (5.8)	0.163
COPD, *n* (%)	31 (14.5)	4 (5.3)	27 (19.6)	0.004
Cancer, *n* (%)	23 (10.7)	10 (13.2)	13 (9.4)	0.398

* 1 missing value. ^#^ 2 missing values. Abbreviations: AMI: acute myocardial infarction; BMI: body mass index; CAD: coronary artery disease; CKD: chronic kidney disease; COPD: chronic obstructive pulmonary disease; CR: cardiac rehabilitation; IQR: interquartile range; PAD: peripheral artery disease; SD: standard deviation.

**Table 2:** Heart failure-related clinical features of the studied population according to participation in a cardiac rehabilitation program.

Heart Failure-Related Clinical Features	Overall (*n* = 214)	Enrolled in CR (*n* = 76)	Not Enrolled in CR (*n* = 138)	*p*-Value
Etiology				
Ischemic, *n* (%) *	65 (30.7)	21 (27.6)	44 (32.4)	0.475
Non-ischemic, *n* (%) *	147 (69.3)	55 (72.4)	92 (67.6)	0.475
NYHA functional class				
I-II, *n* (%) ^#^	181 (88.7)	64 (86.5)	117 (90.0)	0.446
III-IV, *n* (%) ^#^	23 (11.3)	10 (13.5)	13 (10.0)	0.446
NT-pro-BNP (pg/mL), median (IQR)	716 (268–2064)	713 (230–1588)	732 (274–2818)	0.567
LVEF (%), median (IQR)	30 (30–39)	30 (30–37)	30 (30–40)	0.717
PASP (mmHg), median (IQR)	33 (26–44)	30 (24–41)	34 (27–45)	0.304
Mitral regurgitation				
Absent/mild, *n* (%)	158 (73.8)	54 (71.1)	104 (75.4)	0.492
Moderate-severe, *n* (%)	56 (26.2)	22 (28.9)	34 (24.6)	0.492

* 2 missing values. ^#^ 10 missing values. Abbreviations: CR: cardiac rehabilitation; IQR: interquartile range; LVEF: left ventricular ejection fraction; NT-pro-BNP: N-terminal pro-B-type natriuretic peptide; NYHA: New York Heart Association; PASP: pulmonary artery systolic pressure; SD: standard deviation.

**Table 3:** Pharmacologic treatment and cardiac implantable electronic devices of the studied population according to participation in cardiac rehabilitation program.

Pharmacologic Treatment	Overall (*n* = 214)	Enrolled in CR (*n* = 76)	Not Enrolled in CR (*n* = 138)	*p*-Value
Beta-blocker, *n* (%)	200 (93.5)	74 (97.4)	126 (91.3)	0.146
ACE-I/ARB/ARNI, *n* (%)	199 (93.0)	73 (96.1)	126 (91.3)	0.193
ACE-I/ARB, *n* (%)	147 (68.7)	47 (61.8)	100 (72.5)	0.109
Sacubitril/valsartan, *n* (%)	52 (24.3)	26 (34.2)	26 (18.8)	0.012
MRA, *n* (%)	163 (76.2)	64 (84.2)	99 (71.7)	0.040
SGLT2-I, *n* (%)	90 (42.1)	35 (46.1)	55 (39.9)	0.379
Loop diuretic, *n* (%)	142 (66.4)	49 (64.5)	93 (67.4)	0.666
**Cardiac Implantable Electronic Devices (CIED)**				
ICD, *n* (%)	59 (27.6)	31 (40.8)	28 (20.3)	0.001
CRT, *n* (%)	30 (14.0)	16 (21.1)	14 (10.1)	0.028

Abbreviations: ACE-I: angiotensin-converting-enzyme inhibitor; ARB: angiotensin II receptor blocker; ARNI: angiotensin receptor-neprilysin inhibitor; CIED: cardiac implantable electronic devices; CR: cardiac rehabilitation; CRT: cardiac resynchronization therapy; ICD: implantable cardioverter defibrillator; MRA: mineralocorticoid receptor antagonist; SGLT2-I: sodium-glucose cotransporter-2 inhibitor.

**Table 4:** Clinical determinants of non-enrollment in a CR program of patients with heart failure with reduced ejection fraction—univariate analysis and respective crude odds ratio (OR).

Clinical Determinants	Univariate Analysis		
	Crude OR	95% CI	*p*-Value
Age (years)	1.04	(1.01–1.07)	0.004
Higher education level (>4th grade)	2.76	(1.47–5.21)	0.002
Hypertension	1.74	(0.99–3.06)	0.056
Stroke	2.41	(0.86–6.70)	0.093
COPD	4.38	(1.47–13.0)	0.008
Sacubitril/valsartan	0.45	(0.24–0.84)	0.013
MRA	0.48	(0.23–0.98)	0.043
ICD	0.37	(0.20–0.68)	0.002
CRT	0.42	(0.19–0.92)	0.031

Abbreviations: CI: confidence interval; COPD: chronic obstructive pulmonary disease; CR: cardiac rehabilitation; CRT: cardiac resynchronization therapy; ICD: implantable cardioverter defibrillator; MRA: mineralocorticoid receptor antagonist; OR: odds ratio.

**Table 5:** Clinical determinants of non-enrollment in a CR program of patients with heart failure with reduced ejection fraction—multivariate analysis and respective adjusted odds ratio (OR).

Clinical Determinants	Multivariate Analysis		
	Adjusted OR	95% CI	*p*-Value
Age (years)	1.04	(1.01–1.07)	0.005
Higher education level (>4th grade)	3.31	(1.63–6.70)	0.001
Hypertension	1.38	(0.71–2.69)	0.338
Stroke	3.29	(1.06–10.27)	0.040
COPD	4.82	(1.53–15.16)	0.007
Sacubitril/valsartan	0.56	(0.27–1.15)	0.113
MRA	0.54	(0.24–1.21)	0.135
ICD	0.37	(0.19–0.73)	0.004
CRT	0.53	(0.17–1.58)	0.253

Abbreviations: CI: confidence interval; COPD: chronic obstructive pulmonary disease; CR: cardiac rehabilitation; CRT: cardiac resynchronization therapy; ICD: implantable cardioverter defibrillator; MRA: mineralocorticoid receptor antagonist; OR: odds ratio.

**Table 6:** Barriers to cardiac rehabilitation enrollment of patients with heart failure with reduced ejection fraction according to the Cardiac Rehabilitation Barriers Scale (CRBS) questionnaire.

Barriers to CR Program Enrollment (According to CRBS Questionnaire)	Patients Not Enrolled in CR (*n* = 138)
I did not attend a cardiac rehabilitation program because:	n (%)
1. … of distance (e.g., not located in your area, too far to travel)	12 (8.7)
2. … of cost (e.g., parking, gas)	3 (2.2)
3. … of transportation problems (e.g., access to car, public transportation)	-
4. … of family responsibilities (e.g., caregiving)	2 (1.4)
5. … I didn’t know about cardiac rehab (e.g., doctor didn’t tell me about it)	7 (5.1)
6. … I don’t need cardiac rehab (e.g., feel well, heart problem treated, not serious)	-
7. … I already exercise at home, or in my community	2 (1.4)
8. … severe weather	1 (0.7)
9. … I find exercise tiring or painful	2 (1.4)
10. … travel (e.g., holidays, business, cottage)	3 (2.2)
11. … of time constraints (e.g., too busy, inconvenient class time)	1 (0.7)
12. … of work responsibilities	4 (2.9)
13. … I don’t have the energy	1 (0.7)
14. … other health problems prevent me from going:	
14.1. musculoskeletal problems	20 (14.5)
14.2. other health problems	19 (13.8)
15. … I am too old	-
16. … my doctor did not feel it was necessary	42 (30.4)
17. … many people with heart problems don’t go, and they are fine	-
18. … I can manage my heart problem on my own	-
19. … I think I was referred, but the rehab program didn’t contact me	1 (0.7)
20. … it took too long to get referred and into the program	-
21. … I prefer to take care of my health alone, not in a group	-
22. Other reason(s) for not attending a cardiac rehabilitation program:	
22.1. COVID-19 pandemic	2 (1.4)
22.2. concomitant pulmonary rehabilitation	1 (0.7)
22.3. patient refusal—no reason	15 (10.9)

Abbreviations: CR: cardiac rehabilitation; CRBS: Cardiac Rehabilitation Barriers Scale; HFrEF: heart failure with reduced ejection fraction.

## Data Availability

The data that support the findings of this study are available from the corresponding author upon reasonable request.

## Abbreviations

CIED	Cardiac Implantable Electronic Devices
COPD	Chronic Obstructive Pulmonary Disease
CR	Cardiac Rehabilitation
CRBS	Cardiac Rehabilitation Barriers Scale
CRT	Cardiac Resynchronization Therapy
ESC	European Society of Cardiology
ICD	Implantable Cardioverter Defibrillator
LVEF	Left Ventricular Ejection Fraction
HF	Heart Failure
HFrEF	Heart Failure With Reduced Ejection Fraction
NT-pro-BNP	N-Terminal Pro-B-Type Natriuretic Peptide
NYHA	New York Heart Association
QOL	Quality of Life
